# Underwater Multi-Vehicle Trajectory Alignment and Mapping Using Acoustic and Optical Constraints

**DOI:** 10.3390/s16030387

**Published:** 2016-03-17

**Authors:** Ricard Campos, Nuno Gracias, Pere Ridao

**Affiliations:** Computer Vision and Robotics, University of Girona, Girona 17071, Spain; ngracias@eia.udg.edu (N.G.); pere@eia.udg.edu (P.R.)

**Keywords:** SLAM, multi-vehicle, mapping, underwater

## Abstract

Multi-robot formations are an important advance in recent robotic developments, as they allow a group of robots to merge their capacities and perform surveys in a more convenient way. With the aim of keeping the costs and acoustic communications to a minimum, cooperative navigation of multiple underwater vehicles is usually performed at the control level. In order to maintain the desired formation, individual robots just react to simple control directives extracted from range measurements or ultra-short baseline (USBL) systems. Thus, the robots are unaware of their global positioning, which presents a problem for the further processing of the collected data. The aim of this paper is two-fold. First, we present a global alignment method to correct the dead reckoning trajectories of multiple vehicles to resemble the paths followed during the mission using the acoustic messages passed between vehicles. Second, we focus on the optical mapping application of these types of formations and extend the optimization framework to allow for multi-vehicle geo-referenced optical 3D mapping using monocular cameras. The inclusion of optical constraints is not performed using the common bundle adjustment techniques, but in a form improving the computational efficiency of the resulting optimization problem and presenting a generic process to fuse optical reconstructions with navigation data. We show the performance of the proposed method on real datasets collected within the Morph EU-FP7 project.

## 1. Introduction

Cooperative surveying using multiple autonomous vehicles has become an ubiquitous topic in the robotics community. Among the advantages that these methods provide, the most relevant is the ability to parallelize the survey of a given area by using multiple robots, thereby opening the door to large area mapping in less time than when using a single robot. A possible use of multiple robots may be to divide the area of interest into smaller parts that each vehicle can cover independently [1]. In this article, however, we focus on the use of a group of vehicles which, with the help of a moving communications network, behave as a single entity navigating within formation control. More precisely, we aim at performing an optical survey using a formation of underwater robots in order to obtain a three-dimensional (3D) map of the interest area using monocular vision.

Formations of multiple robots have been used in aerial and terrestrial applications [2,3,4], however the burden posed by communication prevents the formation control scheme proposed in most of those approaches to be used with Autonomous Underwater Vehicles (AUVs). Underwater acoustic communications are challenging due to high latency, low bandwidth and throughput and low reliability when compared to in-air communication. Thus, the amount of data passing between underwater vehicles must be kept to a minimum.

Global navigation is another challenge for underwater vehicles because global positioning system (GPS) signals are attenuated by water. Often, global positioning is obtained through a support surface vehicle, coupled with Ultra Short Base Line (USBL) systems to communicate the location to the underwater vehicles on the formation [5]. But USBL systems are costly, and the information is difficult to pass to great depths. Relative range measurements between underwater vehicles are normally used to bound their drift within the formation.

Ideally, to alleviate the costs of these vehicles, each robot in a formation should carry only the minimal set of sensors required to fulfil their role. This means that the on-vehicle processing should also be kept to a minimum. For these reasons, simpler monocular cameras may be preferred over more complex and expensive stereo sets.

**Figure 1:**
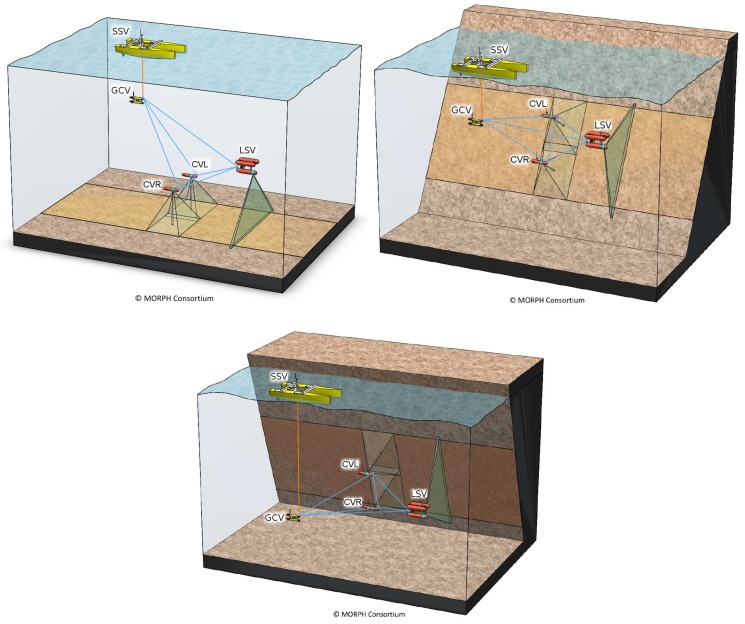
The formation envisioned in the Morph EU-FP7 project. Note in the 3 possible configurations shown how the Morph formation can vary depending on the shape of the area of interest being surveyed. Figures courtesy of the Morph consortium.

For all the above-mentioned reasons, the formation is supposed to work at control level. The trajectories of the individual vehicles are corrected on demand, based on the acoustic measures passed between the formation and basic directives. This means that each vehicle has its own dead reckoning trajectory incorporating all the navigation sensors on-board, such as the Inertial Measurement Unit (IMU), the Doppler Velocity Log (DVL), *etc*. The trajectories of individual vehicles are not corrected with respect to that of the other vehicles in the formation. A good example of this type of formation is that proposed by the Morph EU-FP7 project [6], visible in Figure 1. In this case, the formation is not fixed, since it needs to react to the terrain and adapt to obstacles, which may include flat areas, or more complex rugged terrains. During the mission execution, all vehicles keep a desired formation with respect to the leader vehicle (LSV in Figure 1). However, after recovering the vehicles, the on-board computed trajectories stored on each robot do not resemble the formation performed in real time. Obviously, this complicates further processing to be applied on the collected data.

In this paper, we propose a method to correct the trajectories of individual underwater vehicles used in formation, so that their recovered global positions can be used to obtain a georeferenced 3D map of the area using the monocular imagery collected by each vehicle. A well known problem of using monocular instead of stereo cameras is that the resulting 3D maps constructed from pure optical data suffers the problem of scale ambiguity [7]. In this paper, we tackle the problem of giving a proper georeferencing to these 3D models (and consequently, a proper scale) by fusing the pure optical-based trajectory with the metric trajectory followed by the robot.

We start by introducing the related work on the topics described, to then define our optimization framework using the USBL and range fixes between robots collected during the mission to correct the dead-reckoning trajectories offline. Next, we propose a method to georeference a pure optical 3D reconstruction by means of adapting it to the vehicle navigation readings. Finally, we present the results of applying the methods to real datasets. The main symbols and acronyms used throughout this paper are presented in Appendix A.

## 2. Related Work

Problems related to multiple-vehicle localization and/or mapping are a hot topic in the robotics community nowadays. Many developments in cooperative unmanned vehicles can be found in recent literature for the case of land vehicles [2,8], aerial vehicles [3,4,9] or even a combination of both [10].

Multiple-vehicle localization is more complex in the underwater environment, however, given severe constraints on communications bandwidth, which means the amount of information exchanged between robots must be minimized. The control architecture of such vehicles can be presented in two forms: either the task is split into several vehicles and they just communicate the minimum amount of information to relocalize themselves, or they work in formation control, performing a task cooperatively.

On the one hand, cooperative localization algorithms deal with the inter-localization of the vehicles when they have the ability to communicate and measure the relative positions between each other [1,8,11,12,13]. That is, individual local estimations of localization within each vehicle are corrected when possible (*i.e.*, when two robots can measure and pass some sort of relative measurement between them), and they further correct their localization with respect to the others. Consequently, this provides a global localization consistent within different robots.

On the other hand, there are applications where having a group of robots navigating cooperatively within a formation is more desirable (e.g., the inspection of a pipeline [14]). The leader-followers formation, where one of the vehicles is followed by the rest, is one of the most commonly used approaches [14,15,16,17]. It provides the advantage of only having to broadcast the necessary information of the leader to the others. In the cases where the leader is a surface vehicle, the navigation error can be bounded by USBL [6] or even more closely by GPS [5,18,19]. When all the vehicles are underwater, however, most modern approaches use scalar ranges [16,19]. Having the vehicles equipped with underwater acoustic modems, ranges (*i.e.*, Euclidean distances) are the simplest and easiest hint of location, as a data packet sent between two vehicles with adequate time stamping and assuming known sound velocity in water provides this information directly.

The problem of localizing multiple robots is obviously closely related to the problem of mapping, and consequently many Simultaneous Localization and Mapping (SLAM) approaches have been defined in this sense. The natural extension from single to multiple-vehicle SLAM is to model the state of the group as a single system. These approaches are referred to as centralized SLAM solutions, and they have shown useful for specific applications [20,21,22]. However, it is clear that these systems do not scale well with the complexity of the surveys and require a proper communication channel between vehicles. To overcome these limitations, the distributed SLAM algorithms were created. In distributed SLAM, each vehicle builds local maps and trajectories, and they devise strategies to keep these local approximations within a global coherence with respect to the maps and trajectories on the other vehicles using the passing of messages between vehicles [23,24,25,26,27,28,29,30,31]. Given limited communications underwater, distributed approaches are more likely to be useful for underwater applications. Among others, optical sensors are commonly used for the landmark recognition task and even as navigation sensors [3,32]. However, high-resolution maps are not usually the target of this type of approach, and consequently there are few references in the literature for the case of multi-vehicle high-resolution optical mapping either for 2D [33], 2.5D [34] or full 3D [35,36].

After this brief revision of the state of the art, we can now position our method with respect to the other approaches. Our trajectory optimization and mapping falls within the multi-vehicle SLAM problem. Unlike other multi-vehicle SLAM approaches, however, we focus on the post-processing of the data, as we assume the problem of real-time navigation of the multi-vehicle structure is solved at control level. Our approach focuses on recovering the real trajectories from the internally computed navigation within each robot, which are not aware of the changes induced by the inter-robot measurements, and hence are bound to drift. Thus, while the real-time performance of the control architecture leads to a successful survey of a site in formation, the resulting georeferenced data collected after the mission is not corrected. Although our method can be classified as a SLAM problem, we differentiate from the other methods in that we divide the process in two steps: the first part takes care of the proper correction of the localization of multiple vehicles alone. It is just in the second step where we take into account the simultaneous optimization of localization and mapping for the case of monocular cameras. Our approach could be considered a centralized SLAM solution, where we take advantage of all the data we gathered during the mission to build high-resolution optical georeferenced maps of the surveyed area of interest.

## 3. Overview

The dead-reckoning trajectories logged by each individual vehicle in the formation need to be corrected with all the information gathered during the actual mission to then use the monocular optical constraints to map the area of interest.

In multi-AUV structures that need to navigate cooperatively, the formation control of the vehicles must be performed by some sort of message passing between vehicles that allows them to correct their poses with respect to that of other vehicles in the formation. The most commonly used types of inter-vehicle messages are simple range measurements or USBL fixes. In the first case, ranges are extracted from time-of-flight computation of messages passed between the on-board modems and known sound velocity in water. In the second case, USBL systems mounted on the vehicles, provide more complex fixes in the form of a relative translation measurement in ***X***, ***Y*** and ***Z*** directions with respect to an origin (located, in this case, in one of the moving vehicles). Thus, both USBL fixes and range measurements are important to act as *anchors* to the trajectory correction, so that they are in agreement with those measures. Note that, due to the aforementioned communication constraints, these corrections are very sparse when compared to the frequency at which the internal navigation of each vehicle is updated by on-board sensors. Also, these measures are usually noisy, and they are not accurate enough to build an optical map of the area. Trajectories recovered using optical data are usually smoother and less noisier, allowing also to create a map of the surveyed area.

**Figure 2:**
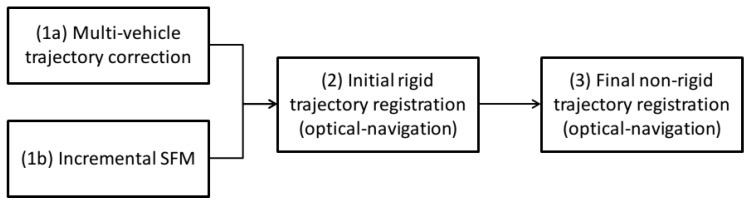
The processing pipeline presented in this article: Steps (1a), (1b), (2) and (3) correspond to Section 4, Section 5.1, Section 5.2.1 and Section 5.2.2 respectively. Steps (1a) and (1b) can run in parallel, as they do not need to share information.

The aim of this article is to propose a generic framework to optimize trajectories of robots based on the most used acoustic measures underwater, as well as a generic tool to obtain optical 3D maps with inexpensive monocular cameras. Moreover, integrating both navigation and optical constraints during the map construction process resolves the up-to-scale limitation of monocular optical reconstruction systems, obtaining a metric georeferenced 3D map of the surveyed area. The processing pipeline is illustrated in Figure 2 and described in subsequent sections.

## 4. Multi-Vehicle Trajectory Correction

To correct the trajectories of the vehicles in formation, we followed the common approach and formulated the problem using a factor graph. In factor graphs, the problems are modelled as a bipartite graph composed of two types of nodes. On one hand, we find the *poses* $X^{*}$, representing the variables to optimize, which in our problem correspond to the actual poses $x_{t}^{v}$ of vehicle *v* at time *t*. We represent them in the more general term using 6 degrees of freedom (DOF). In our formulation, the 3D pose is described by 6 scalars $x_{t}^{v}=\left(x,y,z,q_{x},q_{y},q_{z}\right)$, being (x,y,z) local coordinates in meters from a North-East-Down reference frame, and $\left(q_{x},q_{y},q_{z}\right)$ being the axis of normalized unit quaternions, as proposed in [37]. This compact representation can be converted to a 4×4 transformation matrix. On the other hand, we find the *factors*, encapsulating the measurements and relating them to the current state of the poses in the graph. In other words, each of the factors encapsulate part of the error function to minimize. Thus, we want the poses to be as much in concordance as possible to the input measurements.

Pose and factors together compose the global error function to minimize. Given a proper definition of the poses and factors of the graph representation, many non-linear solvers may be used to minimize the resulting cost function. We decided to extend the g^2^o framework [37] to take advantage of the many different solvers already available in the package. More precisely, our minimization has the following form:

(1) $$X^{*}=\underset{X}{\mathrm{argmin}}\;\sum_{X}\left\{A+O+R+U\right\}$$

where A, O, R and U are the terms to minimize, each one associated with a given available measure. We describe in the following sections each of these terms.

### 4.1. Absolute Constraints

These constraints represent the reliability of each vehicle’s own dead reckoning, in global positioning terms:

(2) $$A=\sum_{v,t}{∥x_{t}^{v}-a_{t}^{v}∥}_{\Sigma_{a_{t}^{v}}}^{2}$$

where $a_{t}^{v}$ is a pose vector measurement. Note that in Equation (2), and in further terms of the minimization in Equation (1) to be introduced later on, we use the Mahalanobis distance ${∥e∥}_{\Sigma }^{2}$, where Σ represents the covariance matrix of the measure.

These factors can be set with a very low covariance when a GPS-enabled surface vehicle is part of the formation. Even in that case, however, the factors are also useful to give a weight to the reliability of each of the global trajectories of each vehicle, or even a specific part of those measurements. For instance, by tuning the covariances imposed for each measurement, we could give small covariance to the Z component, since depth readings are normally accurate underwater (given the use of pressure sensors), and leave a high variance to the remaining components, so that they are not largely restraining the poses in the optimization.

Furthermore, these constraints, in conjunction to those explained in Section 5 will form the basis of our method to recover a georeferenced 3D model from a pure optical reconstruction.

### 4.2. Relative Constraints

Relative priors refer to the odometry measures between two sequential poses in the trajectory of a single vehicle. Thus, given poses $x_{t-1}^{v}$ and $x_{t}^{v}$, the relative constraint comes from the difference between the measured odometry and that implied by the given state of the pose nodes in the graph. It has the following form:

(3) $$O=\sum_{v,t}{∥x_{t-1}^{v}⊕o_{t}^{v}-x_{t}^{v}∥}_{\Sigma_{o_{t}^{v}}}^{2}$$

where $o_{t}^{i}$ is a relative pose measurement, and ⊕ is the compounding operator as defined by Smith *et al.* [38].

### 4.3. Range Constraints

As previously mentioned, range measurements between two vehicles equipped with acoustic modems can easily be computed using the known speed of sound in water and the time required for a message to travel from the emitting vehicle to the receiver. Although simple, this information provides a distance restriction between vehicles:

(4) $$R=\sum_{v,w,t}{∥\mathrm{dist}\left(x_{t}^{v},x_{t}^{w}\right)-r_{v,w,t}∥}_{\Sigma_{r_{v,w,t}}}^{2}$$

$\mathrm{dist}(x_{a},x_{b})$ being the Euclidean distance between the two poses in the graph (note how, in this case, they correspond to two different vehicles *v* and *w*), and $r_{v,w,t}$ being the measured scalar range between vehicle *v* and *w* at time *t*.

### 4.4. USBL Constraints

Vehicles equipped with a USBL sensor can provide relative translation constraints at a given time with respect to another vehicle:

(5) $$U=\sum_{v,w,t}{∥\mathrm{disp}\left(x_{t}^{v},x_{t}^{w}\right)-u_{v,w,t}∥}_{\Sigma_{u_{v,w,t}}}^{2}$$

where $u_{t}^{v,w}$ is the 3-element vector describing the measured relative displacement between vehicles *v* and *w* at time *t*, and $\mathrm{disp}(x_{a},x_{b})$ is the translation part of the compounded pose $x_{a}⊕x_{b}$.

## 5. Georeferenced Monocular Optical Mapping

Given the optimized navigation trajectories obtained from the minimization posed in the section above, our final objective is to reconstruct the observed scene in 3D using all the vehicles in the formation equipped with cameras.

A common approach for 3D mapping is to rely on stereo cameras. By having the known extrinsic geometry between cameras, the recovered reconstructions are directly metric, which eases further processing of this data by third parties. Although the use of stereo cameras is extensive, monocular cameras provide a less-expensive, yet effective, alternative. The major problem with monocular cameras is that, if no additional information is included in the pipeline, the models and camera trajectories reconstructed from the images alone are correct only up to an unknown scale factor.

There is a vast literature in SLAM posing the problem as a bundle adjustment minimization. with bundle adjustment, each of the points forming the sparse structure of the observed scene, as well as the camera poses are optimized using non-linear least squares techniques [34]. However, we found that adding the navigation constraints during monocular reprojection error-based techniques may be too restrictive in some cases. The navigation poses are usually not accurate enough with respect to the resolution expected for imaging sensing. Thus, when initializing the sparse structure from those poses, the points tend to attain a large error in the reprojection sense, which results in a large number of 3D points finding a wrong stable minimum and being discarded. Thus, our approach starts by computing an incremental Structure-from-Motion (SFM) from optical data solely, possibly generating multiple reconstructions in parts of poor visibility, and then registering them with the navigation constraints optimized as explained previously in Section 4. The next two sections detail this method.

### 5.1. Structure and Motion Estimation

**Figure 3:**
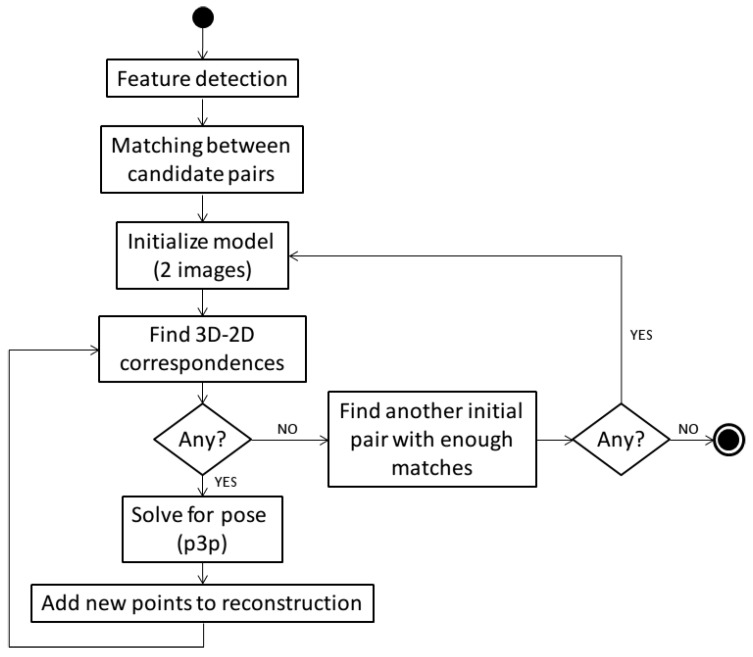
Flux diagram of the Structure-from-Motion pipeline used to construct the initial up-to-scale 3D models from optical data only. After feature detection and matching, a 2-view model is used to initialize the structure, which is then progressively updated by adding new cameras and 3D points using 3D-2D matching and resectioning.

To reconstruct the scene and the motion of the camera from pure optical data we use a variant of the incremental SFM technique described in Nicosevici *et al.* [39]. We assume known intrinsics for the camera, and the pipeline (Figure 3) follows that of other commonly-used SFM methods [40,41].

In short, after extracting the features for each image (we use a GPU implementation of SIFT feature detection/description [42,43]), we compute all the matches between candidate pairs using RANSAC [44] with the essential matrix as the model for the image motion constraints. Note that in order to find the set of candidate matching image pairs to test, we can take advantage of the prior knowledge of the poses of the vehicles in the optimized trajectories to restrict the search for matches. Thus, given a pose associated to a given image, we consider as potential sequential and non-sequential matching pairs all those other images whose corresponding vehicle pose is within a given radial neighbourhood from the test one.

Once the pair-wise geometry has been established, we resort to an incremental reconstruction of both structure and camera poses (*i.e.*, trajectories). First, given the pair-wise matches, the features are tracked across different views. Then, starting from a sufficiently reliable image pair containing enough matches, we compute an initial 3D model using pure epipolar geometry. Since we tracked the features, we can know which is the image that sees most of the reconstructed 3D points. We then infer the 3D-2D relations between features in the model and in the images and use the p3p method [45] to obtain the camera pose (again inside a RANSAC procedure to account for outliers in the association). Once the camera pose is recovered, new 3D points can be generated and a bundle adjustment step is performed. Note that this process is iterated until no more cameras see enough points in the current 3D model. Then, if there still exist some image pairs sharing enough matches, then we can generate another initial model and start the incremental motion/structure building steps.

The incremental nature of the method is needed because the pair-wise geometry is expected to contain outliers. By adding the next camera with more reconstructed points at each step we greatly reduce the chances of adding a wrong camera position (and, consequently, 3D outlier points) in the process.

### 5.2. Georeferencing the Reconstruction

Once we have the scene and camera trajectory reconstructed from optical data alone, possibly in the form of multiple unconnected reconstructions, we need a way of georeferencing the reconstructions according to the real scene, also recovering a metric model/trajectory. Thus, we are aiming to register the previously optimized georeferenced navigation obtained after applying the method in Section 4 (and hereinafter simply referred to as navigation trajectory) with that obtained from the images only. Note that, by registering both trajectories, we are also registering the associated 3D models.

Since the image-based reconstruction recovers the camera trajectory, we need to relate the navigation trajectory of the vehicle frame to the trajectory followed by the camera frame, so that we can register both. In our case, the transformation between the vehicle and the camera frames is assumed to be rigid and known. Thus, we can easily relate the trajectory with respect to the camera frame to that with respect to the vehicle frame and vice-versa.

The typical formulation of the problem would be the use of bundle adjustment-like techniques. In this sense, the absolute constraints presented in Section 4.1 could be used in conjunction with the typical reprojection error functionals [46] for each 3D point in the sparse optical model. However, this would be prohibitively expensive for large datasets, and a good initialization for this problem is of utmost importance in this case. Note that, for moderate-sized datasets, this formulation easily leads to a number of reprojection constraints far larger than the number of navigation constraints, and consequently the minimization is governed by the reprojection errors. This is derived from the fact that the incremental SFM performs periodic bundle adjustment steps, and thus the resulting optical model and trajectory are a minimum in the reprojection error sense. If navigation constraints are imposed, even if they are accurate, they would cause in any case a distortion of this optical trajectory, which is indeed a strong minimum with respect to the reprojection error. Thus, the non-linear solver is expected to converge close to the initial state without taking into account the navigation constraints.

For the above mentioned reasons, we devised a way to encapsulate the trajectory constraints that come from the SFM result and use it inside the optimization framework in a way that is computationally cost-effective and generic. Note that this part is decoupled from both the SFM and the optimization of trajectories on purpose. Therefore, this generic technique can be applied to any existing SFM model which can be related to navigation readings.

The method (corresponding to Steps 2 and 3 in Figure 2) consists of first finding an initial rigid registration between the optical-based extracted camera trajectory and optimized navigation trajectories. Then, we allow non-rigid registration between the camera and navigation trajectories by imposing, at every time instance, the optical constraints in a compact form.

#### 5.2.1. Initial Rigid Trajectory Registration

The registration of both optical and navigation trajectories is a non-rigid registration process. That is, we aim at maintaining the best of both trajectories in the final result; we want the global georeferenced information provided by the navigation trajectory to be merged with the smoothness and local details of the optical one.

The unknown scale factor and the unknown georeferencing for the optical trajectory makes it essential to have a good initial estimation of the transformation. Thus, we infer an initial rigid registration, in the form of a similarity transformation, between optical and navigation trajectories. This translates the problem into finding a proper scale *s*, 3×3 rotation matrix *R* and 3×1 translation vector t that minimizes the differences between the two trajectories.

We relate each image pose to a pose in the navigation by interpolation if needed (*i.e.*, we force a one-to-one mapping between them). We refer to these trajectories, represented by a set of pose vectors, as $X_{\mathrm{Nav}}$ for the navigation (with respect to the camera frame) and $X_{\mathrm{Opt}}$ for the optical trajectory.

Then, we first deal with the trajectories without taking into account the orientation of the vehicles at each frame, and working just with its translation parameters. By making this simplification, we can consider the two trajectories as two point clouds, easing in this way the registration process. To distinguish this trajectory as a point cloud from the original $X_{Y}=\left(x_{Y}^{1},x_{Y}^{2},...,x_{Y}^{n}\right)$, we name it $P_{Y}=\left(p_{Y}^{1},p_{Y}^{2},...,p_{Y}^{n}\right)$. Then, the registration simplifies to:

(6) $$\left(s,R,t\right)=\underset{s,R,t}{\mathrm{argmin}}\;\sum_{i=1}^{N}{∥\left(s\cdot R\cdot p_{Nav}^{i}-t\right)-p_{Opt}^{i}∥}^{2}$$

An approximation of the scale *s* is extracted from the ratio between the diagonal of the bounding box enclosing the navigation trajectory $\mathrm{diag}(P_{\mathrm{Nav}})$ and that of the optical trajectory $\mathrm{diag}(P_{\mathrm{Opt}})$:

(7) $$s=\frac{\mathrm{diag}(P_{\mathrm{Nav}})}{\mathrm{diag}(P_{\mathrm{Opt}})}$$

Then, to find an initial guess for the rotation, we apply the previously found *s* to all the points in $P_{\mathrm{Opt}}$. Given that we have known point-to-point correspondence between the two trajectories, we continue by extracting the centroids, and computing the covariance matrix of the resulting vectors:

(8) $$C=\sum_{i=1}^{N}\left(p_{Nav}^{i}-\mathrm{centroid}\left(P_{\mathrm{Nav}}\right)\right){\left(p_{Opt}^{i}-\mathrm{centroid}\left(P_{\mathrm{Opt}}\right)\right)}^{T}$$

and, given the common SVD decomposition [U,S,V]=SVD(C), we extract the rotation as $R=VU^{T}$.

Finally, the initialization for the translation t is:

(9) $$t=\mathrm{centroid}\left(P_{\mathrm{Nav}}\right)-\left(R\cdot \mathrm{centroid}\left(P_{\mathrm{Opt}}\right)\right)$$

As previously mentioned, the procedure described so far does not take into account the coherence in orientation between the vehicle frames at each pose. Thus, the *s*, *R* and t parameters found so far are used as the initialization of a non-linear optimization minimizing this orientation agreement. To simplify, we leave the scale parameter fixed, and form a compact pose *b* from *R* and t parameters as defined in Section 4. Within this representation, the minimization with respect to the parameters of *b* is defined as follows:

(10) $$b=\underset{b}{\mathrm{argmin}}\;\sum_{x_{Nav},x_{Opt}}∥x_{Nav}⊕b-x_{Opt}∥$$

This problem is non-linear (because of the non-linearity of the rotation part of the poses) and is solved using Levenberg-Marquardt. Note that this problem can also be cast to a pose-graph representation, but given its simplicity, we did not explore this possibility. Finally, note that this procedure must be repeated for each of the sub-models recovered by the SFM step.

#### 5.2.2. Non-Rigid Trajectory Registration

After the initial rigid alignment of the optical trajectory (or trajectories) to the navigation one, we perform a second, more general, non-rigid registration to account for the possible deformations in the optical model, which may be due to calibration errors or drift in parts where no loop closing was detected or possible.

To this end, we pose again the problem as a factor graph, and re-use some of the concepts defined in Section 4. For simplicity of exposition, we show first how the process to register two navigation trajectories would be, to then introduce the differences when one of the trajectories is up to an unknown scale.

As a matter of fact, to register a navigation trajectory into another we could take all the ingredients to solve this problem from Section 4. That is, the graph would be composed of the odometry constraints of the source trajectory, and each pose in this source trajectory would have an absolute constraint with the value of the target trajectory. In this way, during optimization, the relative constraints would take into account the shape of the trajectory, while the absolute constraints would force the trajectory to *move* towards the desired position, all governed by the covariances imposed to the factors.

The main problem we face here is that the optical trajectory lacks the proper scale. That is, we have a trajectory of the vehicle according to the camera, and in some sense we have an odometry measure between nodes in this trajectory. Thus, the constraints to maintain this up-to-scale optical odometry must take into account this lack of the proper scale. To this end, we designed an up-to-scale transformation constraint:

(11) $$O_{\mathrm{uts}}=\sum_{v,t}{∥\mathrm{uts}\_\mathrm{odom}\left(x_{t_{a}}^{v}⊕e_{t_{b}}^{v},x_{t_{b}}^{v}\right)∥}_{\Sigma_{o_{t}^{v}}}^{2}$$

where, $\mathrm{uts}\_\mathrm{odom}\left(x_{t_{a}}^{v}⊕e_{t_{b}}^{v},x_{t_{b}}^{v}\right)$ is the up-to-scale comparison between two poses after applying the up-to-scale transformation $e_{t_{b}}^{v}$ computed between them on the optical reconstruction. In fact, this constraint is quite similar to the odometry one, and is just in the case of comparing the translation part of the poses that we normalize them before comparison. Consequently, we are not comparing translations, but directions. The error function is thus:

(12) $$\mathrm{uts}\_\mathrm{odom}\left(x,y\right)=\left[\begin{array}{c} \frac{{}^{x}x}{\sqrt{{}^{x}x^{2}+{}^{x}y^{2}+{}^{x}z^{2}}}-\frac{{}^{y}x}{\sqrt{{}^{y}x^{2}+{}^{y}y^{2}+{}^{y}z^{2}}}\\ \frac{{}^{x}y}{\sqrt{{}^{x}x^{2}+{}^{x}y^{2}+{}^{x}z^{2}}}-\frac{{}^{y}y}{\sqrt{{}^{y}x^{2}+{}^{y}y^{2}+{}^{y}z^{2}}}\\ \frac{{}^{x}z}{\sqrt{{}^{x}x^{2}+{}^{x}y^{2}+{}^{x}z^{2}}}-\frac{{}^{y}z}{\sqrt{{}^{y}x^{2}+{}^{y}y^{2}+{}^{y}z^{2}}}\\ {}^{x}q_{x}-{}^{y}q_{x}\\ {}^{x}q_{y}-{}^{y}q_{y}\\ {}^{x}q_{z}-{}^{y}q_{z} \end{array}\right]$$

where the preceding superscript of the components of each pose relates to the pose vector they come from. We impose this up-to-scale transformation constraints not only for consecutive images in the sequence, but for all the pair-wise links between poses that have a sufficient relevant number of mutual reconstructed feature matches in the SFM sparse model (we used 100 in all of our experiments).

It is worth noting the parallels between the up-to-scale transformation constraints presented in this paper and the constraints normally presented in global SFM methods [47,48,49,50]. Normally, the pair-wise (or even triplet-wise) epipolar geometry between images is directly used in the minimization. In fact, they attain fast reconstructions essentially by avoiding an incremental procedure. The main concern of these problems is to find correct pair-wise geometry, as this is prone to errors. For this purpose, they normally optimize separately the rotation (which can be reliably estimated even from small baselines) from the translation part for the poses, and make extensive use of heuristics. In our case, since the incrementally constructed model is supposed to be coherent, the up-to-scale constraints added in this step are considered to be free of outliers, and we can account for both rotations and translation directions at once in the non-linear minimization.

To conclude, note that the pose graph to optimize is minimizing the following function:

(13) $$X^{*}=\underset{X}{\mathrm{argmin}}\;\sum_{X}\left\{A+O_{\mathrm{uts}}\right\}$$

where the set of poses $X^{*}$, corresponding to the optical navigation poses, are constrained by absolute measurements, which correspond to the navigation constraints, and by up-to-scale transformation constraints, corresponding to the optical odometry-like measurements.

### 5.3. Further Mapping Pipeline

After obtaining the final georeferenced poses of the cameras, we follow the common multi-view stereo pipeline to obtain a high-resolution textured 3D model. Since these further steps fall out of the scope of the present paper, we use freely available open-source tools for this purpose. First, we densify the point cloud using a multi-view stereo method [51]. Using this denser version of the point cloud, we apply a surface reconstruction method to obtain a triangulated surface presenting a continuous representation of the shape of the scene [52]. Finally, we give texture to each of the triangles in the model using the original images [53].

The end result of these steps is a high-resolution, 3D representation of the area of interest, suitable for further inspection and analysis.

## 6. Results

The Morph EU-FP7 project [6] employs a multi-vehicle formation in which the robots navigate in close range as a group (see Figure 1). Far from being fixed, this formation is required to react and adapt to the shape and obstacles in the terrain. Moreover, it is specifically designed to survey complex 3D scenarios, including highly rugged terrains as well as vertical walls or even overhangs, which provides the applicability of this technology to a wide range of scientific and commercial areas [54].

In the formation described in Morph, a leading vehicle (named the Local navigation and Sonars vehicle, LSV), carrying a multibeam sonar profiler navigates at the forefront of the formation, flying at a “safe” altitude from the seafloor and detecting possible obstacles for two other vehicles (left and right Camera Vehicles CVL and CVR) flying very close to the bottom, with a much higher risk of collision, acquiring images. The communication vehicle (called Global navigation and Communications Vehicle, GCV) is at the back with the main goal of keeping a good communication channel with the surface support vehicle (SSV). The number of vehicles and the shape of formation may change, depending on the task to perform. Additionally, the orientation of the camera on the CVx vehicles in Morph is not fixed, and thus its transformation varies depending on where the focus must be set on the observed area at each moment.

The vehicles used in these experiments are 2 Medusa AUVs [55], playing the role of SSV and GCV, another Medusa or the Girona 500 AUV [56] as LSV, and Seacat AUV [57] and Sparus II AUV [58] as CVL and CVR respectively.

We start this section by giving an overview of the processes described in the paper with a simple example. This dataset was collected during the experiments performed in the harbour of Sant Feliu de Guíxols (Catalunya, Spain) in June 2015. It consists of three vehicles in the Morph formation running close to a wall of this harbour. The leading vehicle (LSV) was followed by both the GCV and the camera vehicle CVR. While the GCV is at the surface (and thus receiving GPS fixes), the LSV and CVR are submerged, with CVR performing an optical survey of a vertical wall of the harbour first (camera rotated $90^{\circ }$ with respect to the gravity vector), and then the seafloor (camera rotated at $0^{\circ }$).

**Figure 4:**
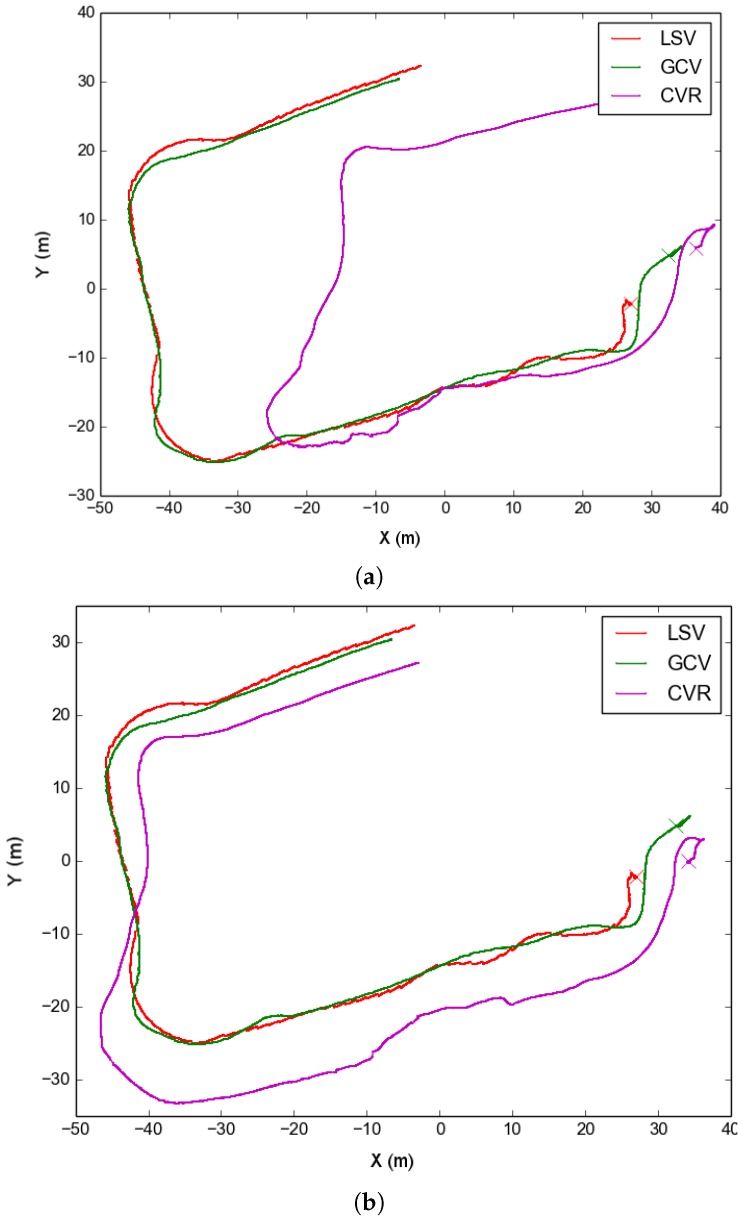

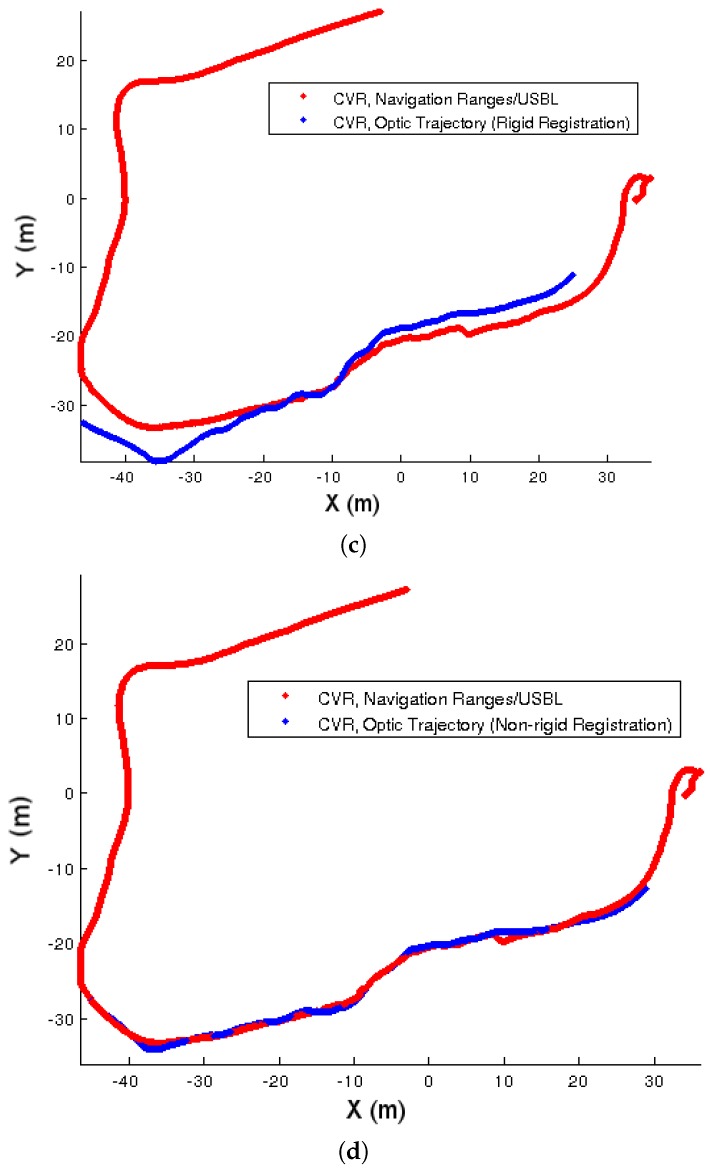
Sant Feliu dataset: trajectories at different steps of the optimization. The original dead reckoning trajectories of each vehicle are shown in (**a**). Then, the navigation trajectories after being optimized using USBL and range measurements are illustrated in (**b**). Finally, the two steps of the optical trajectory optimization are shown in (**c**)—rigid registration—and (**d**)—final non-rigid registration.

The original dead-reckoning trajectories for both LSV and GCV were in agreement, but they differed from the dead reckoning of the Sparus II AUV, which served as CVR (Figure 4a). The disagreement was due to the navigation of the CVR relying too heavily on its DVL, which was inaccurate due to the high relief of the terrain in this survey. After applying the optimization using ranges and USBL fixes, we obtained the result shown in Figure 4b, where the trajectories are now exhibiting a more realistic formation. Then, we reconstructed the observed scene and camera trajectory using SFM, to then register this last one rigidly to the obtained optimized navigation of CVR (Figure 4c). Note that the visibility was not good over all the survey, and that resulted in the optical trajectory being substantially shorter than the navigation one. Just the wall and the part that is close to it after the first turn were reconstructed. Using this vague initialization, we applied the non-rigid registering of this trajectory to resemble the optimized navigation, as shown in Figure 4d. For reference, we include in Table 1 the number of nodes and edges in the pose graph formulation for all the datasets used in this section. It is worth noticing that, while the covariances are not metrically specified, we use the same set of covariances (which act as weights) for all the experiments shown in this section. That is, we give small covariances to the absolute poses of the SSV, a little larger (but still small) to the GCV and LSV, and very large to CVL and CVR.

**Figure 5:**
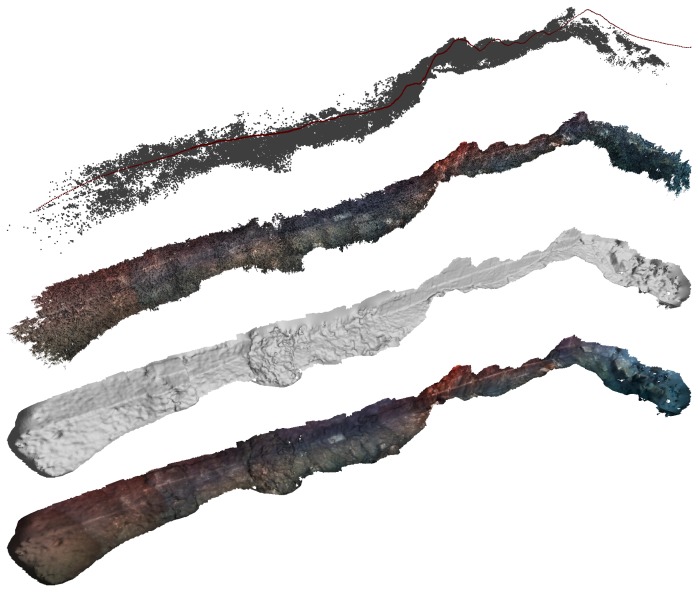
Different steps of the optical reconstruction for the Sant Feliu dataset. From top to bottom, the trajectory of the cameras in red—optimized, corresponding to that of Figure 4d—and the sparse point set resulting from the SFM process, the densified point set, the reconstructed surface and the texture mapping on this last surface.

The optical processing pipeline after the final registration is illustrated in Figure 5. Starting from the trajectory we recovered from the last non-rigid registration step, we re-triangulate the tracks obtained in the SFM step, and we obtain the sparse point set shown in Figure 5 (also the poses for each reconstructed camera are shown using red dots). In subsequent rows of Figure 5, one can observe the denser point set recovered by the multi-view stereo step, which is then used to obtain a surface representation of the scene in the form of a mesh of triangles, where we finally use the texture of the original images to colorize the model.

**Figure 6:**
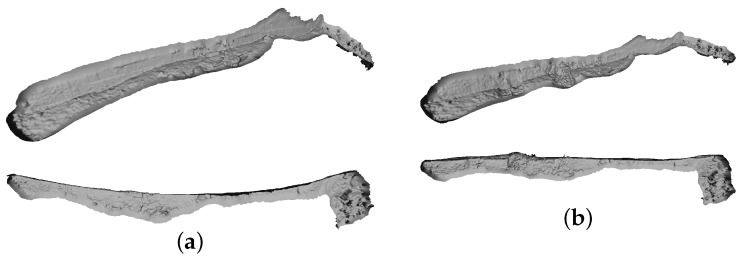
Sant Feliu dataset: slanted (**top** row) and top views (**bottom** row) of both the reconstruction obtained from pure optical data (**a**); and the reconstruction using the non-rigidly registered trajectory (**b**). Note how the unrealistic bending shown in the first is corrected in the second.

Observing a man-made structure allows for the use of visual ground truth. The surveyed wall of the port is known to be vertically flat, so this should be preserved in the resulting model. Figure 6a shows the reconstructed model obtained by just using optical data. Note in the top view how the model shows an unrealistic bending of the wall. This is possibly due to inaccuracies in modelling the distortion parameters of the camera, as well as accumulated drift mainly caused by the fact that this is a sequential survey without loop closings. When we apply the optical registration step, the straightness of the wall is preserved (see Figure 6b). We can also see that the enforcement of the navigation readings can be too restrictive, thus causing deformations in the resulting optical model for some areas. Applying the non-rigid registration to the navigation readings is therefore a trade-off between having a perfect match between the two trajectories, and the preservation of the shape of the model. Thus, the trajectory recovered by the optical data solely needs to be preserved as much as possible while registering it to the navigation readings. This is accounted by the weights imposed to the factors in $O_{\mathrm{uts}}$, which were all set to a constant value in all the experiments shown in this section. In order to mitigate deformations in the model, and as future work, we should further explore the possibility of using different weights for different parts of the trajectory depending on the desired fidelity to the reconstructed model.

**Figure 7:**
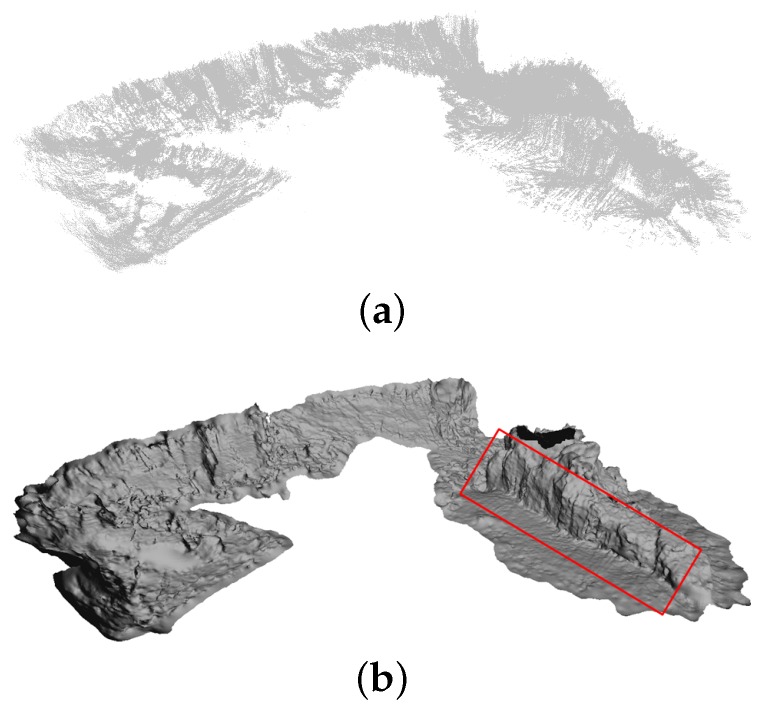
Multibeam-based map of the interest area, objective of the Horta trials 2015, built prior to the experiments. The raw point set in (**a**) was used to build the surface model in (**b**). The marker shows the natural vertical structure, main target of the experiments in Horta for the Morph project.

Additional Morph experiments were performed at the city of Horta (Azores, Portugal) in September 2015. The objective of these experiments was the mapping of an area containing strong 3D relief. A multibeam survey of the area of interest, collected prior to the experiments, is shown in Figure 7 for reference. At this site, the Morph formation was supposed to survey an almost-vertical natural structure (herein referred to as the vertical wall, and marked in Figure 7), and the almost planar area in front of it. Thus, the formation does not have a pre-fixed shape, and must adapt to the properties of the surveyed zone, especially accounting for the positioning of the camera vehicles with respect to the observed surface. Consequently, when surveying the planar area it must keep the CV vehicles in horizontal left/right formation with respect to the line formed by the LSV and GCV, and a vertical equivalent when surveying the wall.

**Figure 8:**
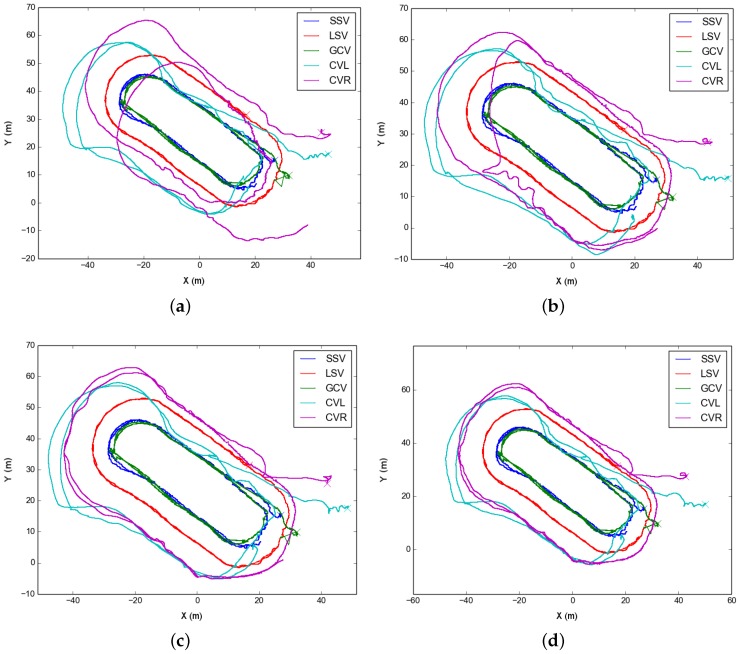
Different trajectories for the Morph Horta dataset at the 9th of September 2015. We show on (**a**) the dead reckoning trajectories of each vehicle; Then, on (**b**,**c**) we can observe the results of optimizing using just ranges or USBL measures respectively; Finally, on (**d**), we show the resulting optimized trajectories accounting for both measurements.

In the first test, on 9 September 2015, the formation performed two concentric oval-shaped trajectories. In this test, the dead reckoning trajectory of CVR substantially differed from the rest of the formation (Figure 8a). This dataset well illustrates the effects of optimizing using just one type of acoustic constraint. For instance, Figure 8b shows the result of optimizing the trajectories using ranges only. Since the range is just an scalar constraint, there is an inherent ambiguity to on which side of the formation the vehicle is located in, and this leads to the strange trajectory shown. On the contrary, as seen in Figure 8c if using the USBL fixes only, the trajectories are mostly well located. This is due to the USBL fixes being a 3D translation constraint, *i.e.*, they are more informative than simpler scalar ranges. Finally, adding both we get a finer correction (Figure 8d).

**Figure 9:**
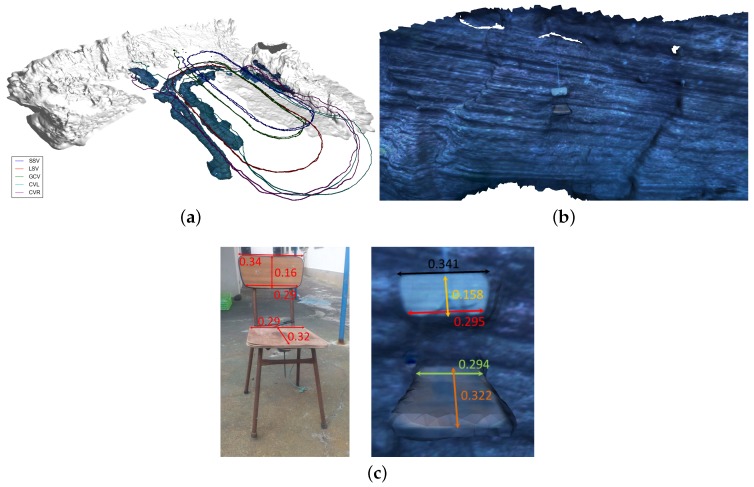
Optical reconstruction for the Morph Horta dataset from the 9th of September 2015. We show the final reconstructed textured model with the optimized trajectories, along with the global bathymetric map for reference in (**a**). Also, we give a close-up view of the reconstructed wall in (**b**), and we compare real-world measurements taken on the ground truth object (the chair) with some taken on the reconstructed model in (**c**).

The optical reconstruction of the Horta survey area can be seen in Figure 9 (with the optimized trajectories of the two robots). Eight models were reconstructed: 4 for CVL and 4 for CVR. Note that, without the registration with the navigation, these unconnected models could not be spatially related to one another. Here we see that for one of the turns there are no reconstructions, and this is because the camera of the robots was tilted to $90^{\circ }$ from the vertical too early and there was nothing to see. The other parts correspond to blurred imagery or the robot being too far from the scene. In order to provide a ground truth reference for the scale of the reconstruction, a known object (a chair) was hung along the vertical structure (as visible in Figure 9b). Dimensions of the chair recovered from the 3D reconstruction were close to the actual values, providing confidence that the correct scale was recovered in the georeferencing step (Figure 9c).

**Figure 10:**
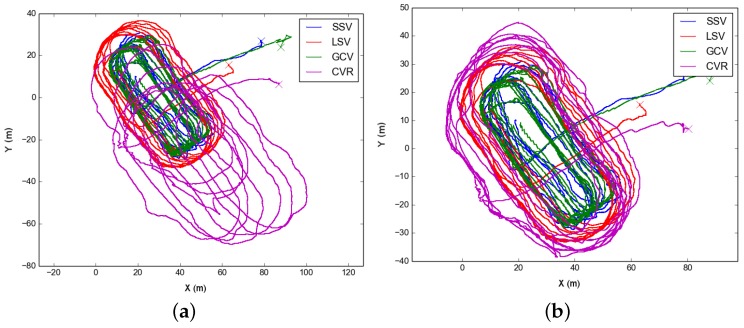
Morph Horta dataset at 11 September 2015: dead reckoning (**a**) and optimized trajectories (**b**).

**Figure 11:**
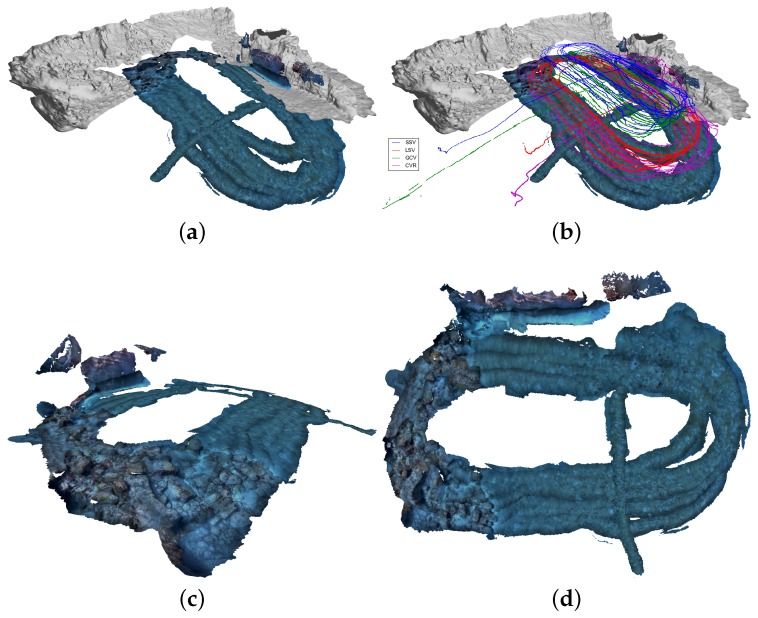
Morph Horta dataset at 11 September 2015: registered optical reconstruction. On (**a**,**b**) we show the reconstructions on the global map, with and without the trajectories; For more details, on (**c**) we show a slanted view highlighting the rocky area in this part; and in (**d**) we show a top overview.

A second test, from 11 September 2015, was collected in the same vicinity as the 9 September data, but over a larger area. In this case, just one camera vehicle was used with the rest of the vehicles in the Morph formation. The dead reckoning trajectories can be seen in Figure 10a, where we can detect the substantial drift of the CVR trajectory. Even with this bad initialization, the optimization using ranges and USBL converges to the correct location for all the vehicles in formation. Moreover, the resulting registered optical reconstruction is also promising (6 models), in the sense that it covers a far larger area, as shown in Figure 11.

## 7. Conclusions

We proposed a multi-vehicle trajectory optimization based on acoustic measurements passed between robots, and we presented a georeferencing method for monocular-based optical reconstructions, both within a pose-graph formulation. The method is generic enough to allow an arbitrary number of vehicles in the formation. We tested its validity with datasets collected during the Morph EU-FP7 project.

The results show that the optimization of trajectories using range and USBL measurements allows the recovery of the proper alignment for the different trajectories corresponding to each vehicle within the same georeferenced frame. Moreover, the registration of optical and navigation trajectories allows not only the proper georeferencing of the data, but also the correction of the possible drift accumulated in a vision-only reconstruction. Finally, the deliberate separation of the optical trajectory registration provides generality to the method, which can be applied to any existing up-to-scale optical reconstruction of a survey that can be related to some sort of navigation data.

As future work, we plan to explore the multi-modal mapping possibilities of the method. For instance, in the Morph project, the leading vehicle in the formation carries a multibeam echosounder. With all the trajectories properly georeferenced, we plan to further fuse bathymetric and optical data in a single map. Another topic to further explore is to properly set the uncertainties in the optimization. Currently, the covariances are used as simple weights, and we need to model them properly in the future.

## Figures and Tables

**Table 1:** Number of poses and factors for the graphs corresponding to the resulting trajectories and models shown in Section 6. Note that the UTSTfs column stands for up-to-scale transformation constraints.

Figure	Pose Vertices					Factors				
	SSV	LSV	GCV	CVL	CVR	Absolute	Relative	Ranges	USBL	UTSTfs
4b	0	16,753	6479	0	12,748	23,232	35,977	291	215	0
4d	0	0	0	0	939	939	0	0	0	12,666
8b	13,976	33,235	14,766	14,053	26,544	61,977	102,569	2859	0	0
8c	13,976	33,235	14,766	14,053	26,544	61,977	102,569	0	1165	0
8d	13,976	33,235	14,766	14,053	26,544	61,977	102,569	2859	1165	0
9	0	0	0	595	638	1233	0	0	0	5733
10b	63,482	168,022	65,853	0	124,014	297,357	421,367	5480	4312	0
11	0	0	0	0	4507	4507	0	0	0	34,396

## Appendix A. List of Acronyms and Symbols

Table A1 shows a list of the main symbols and acronyms used throughout this paper.

**Table A1:** List of main acronyms and symbols.

Acronyms	
2D	Two-dimensional.
3D	Three-dimensional.
SSV	Morph’s Surface Support Vehicle.
LSV	Morph’s Local navigation and Sonars Vehicle.
GCV	Morph’s Global navigation and Communications Vehicle.
CVL	Morph’s Camera Vehicle, on the Left of the formation.
CVL	Morph’s Camera Vehicle, on the Right of the formation.
DVL	Doppler Velocity Log.
IMU	Inertial Measurement Unit.
USBL	Ultra-Short BaseLine.
SLAM	Simultaneous Localization and Mapping.
SFM	Structure From Motion.
**Symbols**	
X	Set of poses to optimize.
e P	Set of 3D points.
A	Absolute constraints.
O	Odometry constraints.
R	Range constraints.
U	USBL constraints.
$O_{\mathrm{uts}}$	Optical odometry constraints.
$\Sigma_{x}$	Covariance of measure *x*.
$x_{t}^{v}$	Pose vector of vehicle *v* at time *t*.
$a_{t}^{v}$	Pose vector measurement for vehicle *v* at time *t*.
$o_{t}^{v}$	Odometry vector measurement for vehicle *v* at time *t*.
$e_{t}^{v}$	Up-to-scale odometry vector measurement for vehicle *v* at time *t*.
$p_{t}^{v}$	3D point representing the translation part of vehicle pose $x_{t}^{v}$.
$r_{v,w,t}$	Range scalar measure between vehicles *v* and *w* at time *t*.
$u_{v,w,t}$	USBL relative translation measure between vehicles *v* and *w* at time *t*.
t	Translation vector.
*s*	Scale.
*R*	Rotation matrix.
$\mathrm{dist}(x_{a},x_{b})$	Euclidean distance between poses $x_{a}$ and $x_{b}$.
$\mathrm{disp}(x_{a},x_{b})$	3-element vector describing the 3D displacement between poses $x_{a}$ and $x_{b}$.
diag(P)	Diagonal length of the bounding box enclosing the point set P.
centroid(P)	Centroid of the point set P.
$\mathrm{uts}\_\mathrm{odom}\left(x_{a},x_{b}\right)$	Up-to-scale error computation between poses.
